# Regulatory T cells in breast cancer drivers of immune suppression and targets for immunotherapy

**DOI:** 10.3389/fimmu.2026.1825637

**Published:** 2026-05-26

**Authors:** Teng Pan, Nanrui Shao, Ruijie Ming, Guanglin Zhou, Yuanyuan Pei, Jinhai Deng

**Affiliations:** 1 Center for Stem Cell and Regenerative Medicine Research, Maternity & Child Healthcare Hospital of Longgang District (Longgang Maternity and Child Institute of Shantou University Medical College), Shenzhen, China; 2 Medical Research Institute of Maternal and Child, Maternity & Child Healthcare Hospital of Longgang District (Longgang Maternity and Child Institute of Shantou University Medical College), Shenzhen, China; 3 Department of Science and Environmental Studies, The Education University of Hong Kong, Hong Kong, Hong Kong SAR, China; 4 Department of Oncology, Chongqing University Three Gorges Hospital, Chongqing, China; 5 School of Medicine, Chongqing University, Chongqing, China; 6 Breast and Thyroid Surgery Department, Maternity & Child Healthcare Hospital of Longgang District (Longgang Maternity and Child Institute of Shantou University Medical College), Shenzhen, China; 7 Baiyunshan Pharmaceutical General Factory, Guangdong Province Key Laboratory for Core Technology of Chemical Raw Materials and Pharmaceutical Formulations, Guangzhou Baiyunshan Pharmaceutical Holding Co., Ltd., Guangzhou, China; 8 Richard Dimbleby Laboratory of Cancer Research, School of Cancer & Pharmaceutical Sciences, King’s College London, London, United Kingdom

**Keywords:** breast cancer, immune regulatory molecules, immunotherapy, regulatory T cells, tumor immune escape

## Abstract

Breast cancer remains one of the most prevalent malignancies and a leading cause of cancer-related death in women worldwide. Increasing evidence indicates that regulatory T cells (Tregs), a specialized subset of CD4^+^ T cells, play a central role in breast cancer initiation, progression, and therapeutic response by shaping an immunosuppressive tumor microenvironment. Tregs originate from both thymic and peripheral differentiation pathways and are characterized by the expression of FoxP3, CD25, CTLA-4, PD-1, and other immune regulatory molecules. In breast cancer, Tregs accumulate within tumor tissues and peripheral blood through chemokine-mediated recruitment, local expansion, and phenotypic conversion, where they suppress antitumor immunity via IL-2 deprivation, inhibitory cytokine secretion, cytolytic activity, metabolic disruption, and checkpoint signaling. Their enrichment is closely associated with tumor immune escape, invasion, metastasis, and poor prognosis. Moreover, emerging therapeutic strategies targeting Tregs, including immune checkpoint blockade, anti-CCR4 antibodies, vaccines, and pathway-modulating agents, have shown promise in restoring antitumor immunity. This review summarizes the origins, phenotypic features, immunosuppressive mechanisms, and clinical relevance of Tregs in breast cancer, and discusses the opportunities and challenges of Treg-centered immunotherapeutic strategies.

## 1 Introduction

Breast cancer remains one of the most prevalent malignancies and a leading cause of cancer-related death in women worldwide, imposing substantial clinical and socioeconomic burdens (1). Accumulating evidence underscores the critical role of the tumor immune microenvironment in governing disease initiation, progression, and therapeutic response (2, 3). Regulatory T cells (Tregs), a specialized FoxP3^+^ CD4^+^ T-cell subset characterized by expression of CD25, CTLA-4, and PD-1, have emerged as central orchestrators of immunosuppression (4, 5). Although indispensable for maintaining peripheral tolerance and preventing autoimmunity under physiological conditions, Tregs are frequently hijacked by breast tumors to establish an immunosuppressive niche that impairs antitumor immunity, facilitates immune escape, and contributes to therapeutic resistance (6, 7).

In breast cancer, Tregs accumulate in tumor tissues and peripheral blood through chemokine-mediated recruitment, local expansion, and phenotypic conversion from conventional T cells (7). They suppress effector T cells, natural killer cells, and other antitumor immune populations via multiple non-redundant mechanisms, including IL-2 deprivation, secretion of inhibitory cytokines such as IL-10 and TGF-β, cytolytic activity, metabolic disruption, and checkpoint molecule engagement (8). Elevated Treg infiltration consistently correlates with advanced tumor stage, increased invasion and metastasis, and unfavorable prognosis, underscoring their clinical significance as both prognostic biomarkers and actionable therapeutic targets (9, 10). Consequently, emerging strategies specifically targeting Tregs—such as immune checkpoint blockade, anti-CCR4 antibodies, FoxP3-based vaccines, and pathway-modulating agents—hold considerable promise for reversing immunosuppression and restoring robust antitumor immunity (11). This review summarizes current understanding of Treg origins, phenotypic features, immunosuppressive mechanisms, and clinical relevance in breast cancer, and critically discusses the opportunities and challenges associated with developing Treg-centered immunotherapeutic approaches for improved patient outcomes.

## 2 Treg origins and differentiation

### 2.1 Developmental origins of Tregs

Tregs constitute a functionally specialized and phenotypically heterogeneous subset of CD4^+^ helper T lymphocytes that are essential for maintaining immune tolerance and preventing autoimmunity (12). Based on their developmental origin, Tregs are broadly classified into two major lineages: thymus-derived natural Tregs (tTregs) and peripherally induced Tregs (pTregs) (13). tTregs arise during thymic selection through high-affinity recognition of self-peptide–MHC complexes, acquiring a stable FoxP3^+^ phenotype that enables potent, cell contact–dependent immunosuppression (14). These cells exhibit broad, antigen-non-specific suppressive activity and can directly inhibit the activation, proliferation, and cytokine production of effector T cells independent of classical MHC restriction. In contrast, iTregs differentiate from naïve CD4^+^ T cells in peripheral tissues under tolerogenic conditions, such as exposure to subimmunogenic antigen doses, TGF-β, or retinoic acid (15, 16). Their suppressive function relies predominantly on the secretion of inhibitory cytokines, including IL-10 and TGF-β, and retains antigen specificity, allowing context-dependent modulation of local immune responses (17, 18). This ontogenetic and functional dichotomy underscores the adaptability of the Treg compartment in balancing immune homeostasis across diverse physiological and pathological settings (19). Recent advances in single-cell RNA sequencing (scRNA-seq) have revealed that Tregs within the tumor microenvironment exhibit substantial intratumoral heterogeneity and dynamic transcriptional states (20, 21). Tumor-infiltrating Tregs (TIL-Tregs) display distinct molecular signatures compared with circulating or lymphoid-resident Tregs, reflecting adaptation to local inflammatory, metabolic, and hypoxic conditions (22). These tumor-adapted Tregs frequently upregulate activation and checkpoint-associated molecules, including CCR8, TIGIT, TNFRSF9, and IL1R2, which are associated with enhanced suppressive capacity and tissue residency (23–25). Single-cell analyses further identify multiple functional subclusters, including effector-like Tregs characterized by high activation status and potent immunosuppressive activity, as well as cytokine-responsive subsets that dynamically respond to inflammatory cues within the tumor microenvironment (15, 26). Notably, in breast cancer—particularly in aggressive subtypes such as triple-negative breast cancer (TNBC)—Treg heterogeneity is markedly increased and correlates with immune evasion and therapeutic resistance (21, 27). Hence, Tregs should be viewed as a spectrum of activation-dependent and tumor-adapted states, rather than a uniform lineage defined solely by origin.

### 2.2 Molecular and environmental regulation of Treg differentiation

At the molecular level, the transcription factor forkhead box protein 3 (FoxP3) serves as the master regulator of Treg development and function (28, 29). Despite FoxP3 has traditionally been described as a suppressor of IL-2 transcription, its expression and stability are governed by a complex network of upstream signaling pathways and epigenetic mechanisms (30, 31). Among these, TGF-β/SMAD signaling plays a central role in initiating FoxP3 transcription, as activated SMAD2/3 complexes bind directly to conserved regulatory regions within the FoxP3 locus (32–35). Suppression of the PI3K/AKT/mTOR pathway is critical for Treg differentiation, as it enables the nuclear localization of FoxO transcription factors, which promote FoxP3 expression and Treg lineage commitment (36, 37).

Cytokine-driven signaling further refines Treg expansion and functional specialization. The IL-33/ST2 axis has been identified as a key pathway promoting the accumulation of tissue-resident and tumor-adapted Tregs (38, 39). IL-33 signaling enhances Treg survival, proliferation, and expression of suppressive mediators, particularly in inflamed and tumor-associated tissues (40, 41). This pathway is especially relevant in breast cancer, where stromal and epithelial damage can release alarmins that shape local immune regulation. Additionally, NF-κB signaling, particularly via the c-Rel subunit, contributes to the formation of transcriptional complexes at the FoxP3 promoter and enhancer regions, thereby facilitating Treg induction during immune activation (42, 43). Stable Treg lineage commitment requires epigenetic remodeling of the FoxP3 locus. The conserved noncoding sequence 2 (CNS2), also known as the Treg-specific demethylated region (TSDR), undergoes DNA demethylation, which is essential for maintaining sustained and heritable FoxP3 expression, particularly under inflammatory conditions (44). In contrast, incomplete demethylation of CNS2 is associated with unstable or transient FoxP3 expression, as observed in certain iTreg populations (45–47). Besides, histone modifications and chromatin accessibility changes further cooperate to stabilize Treg identity and function (48). These signaling and epigenetic networks ensure that Tregs maintain their suppressive phenotype despite environmental fluctuations within the tumor microenvironment. Therefore, Treg differentiation is not a linear process but rather a multidimensional program shaped by developmental origin, inflammatory signaling, environmental sensing, and metabolic adaptation, ultimately generating functionally specialized Treg subsets within the breast cancer microenvironment.

## 3 Tregs in breast cancer initiation and progression

### 3.1 Tregs in the breast cancer microenvironment

#### 3.1.1 Chemokine-driven Tregs in breast cancer

Under physiological conditions, the composition and distribution of T-cell subsets in peripheral tissues are tightly regulated to maintain immune surveillance and prevent malignant transformation (49). In breast cancer, however, this homeostatic equilibrium is subverted by tumor-derived signals that actively recruit and retain immunosuppressive populations (50, 51). Tregs constitute a dominant suppressive subset within the tumor microenvironment, playing a central role in facilitating immune escape and disease progression (52). Tregs enrichment established a spatially organized immunosuppressive microenvironment in breast cancer via chemokine-mediated directional migration (53).

The CCL22–CCR4 axis represents a prototypical pathway governing Treg recruitment to breast cancer lesions (54). Tumor cells and tumor-associated macrophages secrete CCL22, which selectively engages CCR4—a G-protein-coupled receptor constitutively expressed at high levels on Tregs—thereby directing their chemotaxis toward tumor sites (11). This ligand–receptor interaction not only promotes Treg infiltration but also supports their local retention and expansion, amplifying the regulatory compartment within the TME (55). Hypoxia-induced chemokine signaling further refines Treg spatial distribution. Inadequate vascularization in rapidly growing tumors generates hypoxic niches that stabilize HIF-1α, which transcriptionally upregulates CCL28 expression. CCL28 preferentially recruits CCR10^+^ Tregs into hypoxic tumor regions, where these cells exhibit enhanced suppressive function and contribute to angiogenesis, thereby coupling immune evasion with tumor progression and metastatic potential (56).

**Figure 1:**
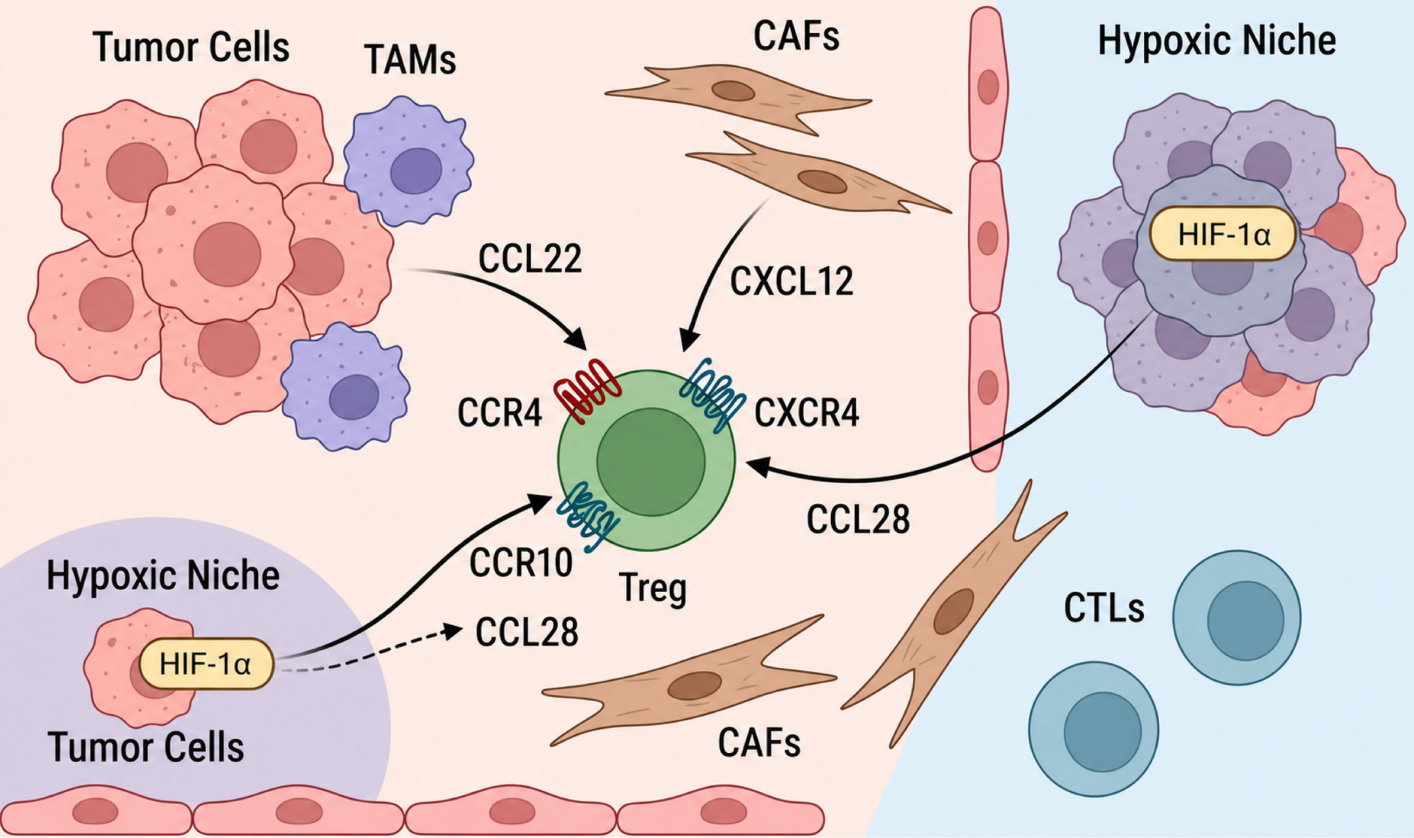
Regulatory T cells recruitment and location in breast cancer.

The CXCL12–CXCR4 axis constitutes another critical regulator of Treg trafficking and microanatomical positioning within breast tumors. CXCL12, abundantly produced by cancer-associated fibroblasts (CAFs), stromal cells, and endothelial compartments, binds CXCR4 on Tregs to promote their localization within tumor-supportive niches (56–58). This signaling axis contributes to the formation of immune-excluded microarchitectures: cytotoxic T lymphocytes (CTLs) are confined to stromal or peripheral regions, whereas Tregs accumulate within the tumor core, physically segregating effector cells from malignant targets and impairing direct tumor-cell killing (59–61). Moreover, CXCL12–CXCR4 signaling is functionally linked to breast cancer metastasis, as it may coordinate the co-migration of tumor cells and immunosuppressive immune subsets toward distant organs such as bone and lung, establishing a pre-metastatic niche permissive for disease dissemination (62) (**Figure 1**).

#### 3.1.2 Local expansion, phenotypic stabilization, and immunosuppressive microenvironment

Local expansion and phenotypic conversion further increase Treg abundance within the breast cancer TME. Cytokines enriched in the local microenvironment, including IL-2, IL-10, and TGF-β, can promote Treg proliferation and induce the conversion of conventional CD4^+^ T cells into induced Tregs, thereby amplifying the intratumoral regulatory compartment (63, 64). TGF-β is particularly important because it promotes FoxP3 expression and stabilizes the regulatory phenotype, whereas IL-2 supports Treg survival through CD25-dependent signaling (31, 65, 66). A memory-like Treg subset characterized by high CD45RO expression and CCR6 positivity also contributes to local Treg persistence and exhibits robust proliferative capacity in response to antigenic stimulation (67, 68). Tumor cells may further reshape the immune composition of the TME by selectively inducing apoptosis in non-Treg effector T-cell populations, resulting in a relative increase in the proportion of Tregs (69, 70). In parallel, activation of co-stimulatory pathways such as ICOS–ICOSL signaling enhances Treg proliferation, survival, and suppressive fitness, thereby strengthening local immune suppression (71, 72). These processes indicate that Treg accumulation in breast cancer is not merely a consequence of passive infiltration, but rather reflects continuous crosstalk among tumor cells, stromal cells, cytokines, and immune checkpoints.

Metabolic stress within the TME also contributes to Treg persistence and functional adaptation. Hypoxia, lactate accumulation, glucose deprivation, and amino acid imbalance collectively impair effector T-cell activity while favoring Treg survival and stability (73, 74). Compared with cytotoxic T cells, Tregs are more capable of adapting to nutrient-restricted and lactate-rich conditions, allowing them to maintain suppressive function in metabolically hostile tumor niches (75, 76). These metabolic advantages enable Tregs to persist where antitumor effector cells become exhausted or dysfunctional (77). Overall, breast tumors promote Treg enrichment through coordinated mechanisms involving chemokine-driven recruitment, local proliferation, phenotypic conversion, selective survival, co-stimulatory signaling, and metabolic adaptation (78). These mechanisms establish a self-reinforcing immunosuppressive niche that limits cytotoxic immune responses, facilitates tumor cell immune evasion, and supports breast cancer progression and metastasis.

### 3.2 Treg-mediated immunosuppression in breast cancer

#### 3.2.1 Direct suppression of antitumor immune activation

The immunosuppressive activity of Tregs is a defining feature of their function within the breast cancer tumor microenvironment (79). Rather than acting through a single pathway, Tregs suppress antitumor immunity through multiple complementary mechanisms that converge on effector T cells, antigen-presenting cells, and innate immune populations (80–82). These mechanisms include cytokine deprivation, direct cytotoxicity, checkpoint-dependent inhibition, and suppression of antigen presentation (83). Through these coordinated processes, Tregs establish an immune-restricted environment that permits tumor cells to evade immune surveillance and continue malignant progression (84, 85). Tregs constitutively express high levels of the IL-2 receptor α chain (CD25) but do not produce IL-2 themselves (86). By competitively consuming IL-2, Tregs deprive effector T cells of this essential growth factor, leading to impaired proliferation, reduced survival, and functional exhaustion (65). This process contributes to the establishment of immune anergy within the tumor microenvironment.

Tregs also suppress antitumor immunity through direct cytotoxic activity, which induce apoptosis of effector T cells, natural killer cells, and antigen-presenting cells by releasing cytolytic molecules such as granzyme A, granzyme B, and perforin (87–89). This mechanism enables Tregs to eliminate immune cells that would otherwise mediate tumor-cell killing or support antigen presentation (90). As a result, the local immune landscape shifts from an immune-activated state toward a tolerogenic state dominated by regulatory and dysfunctional immune populations (91). Checkpoint-dependent suppression represents another central mechanism by which Tregs impair immune activation (92). Tregs constitutively express high levels of CTLA-4, which competes with CD28 for binding to CD80 and CD86 on dendritic cells (93, 94). CTLA-4 can mediate trans-endocytosis of CD80 and CD86, physically removing these co-stimulatory ligands from the surface of dendritic cells (95). This process reduces CD28-mediated co-stimulation in effector T cells and converts dendritic cells into a tolerogenic phenotype with diminished capacity to prime cytotoxic T lymphocyte responses. In parallel, the PD-1/PD-L1 pathway further dampens T-cell receptor signaling (96, 97). Engagement of PD-1 recruits SHP2 phosphatase, which dephosphorylates key proximal signaling molecules, including CD3ζ, ZAP70, and CD28 (98–100). This suppresses downstream PI3K/AKT and MAPK signaling, thereby reducing T-cell proliferation, cytokine production, and cytotoxic function (101, 102). Through these checkpoint-dependent pathways, Tregs suppress antitumor immunity at both the antigen-presentation and effector-response levels (**Figure 1**).

#### 3.2.2 Metabolic, cytokine-mediated, and microenvironmental remodeling by Tregs

Tregs play a critical role in metabolic reprogramming within the tumor microenvironment. A key pathway involves the ectonucleotidases CD39 and CD73, which sequentially hydrolyze extracellular ATP into adenosine. Accumulated adenosine engages A2A receptors on effector T cells, leading to suppression of T-cell activation and cytokine production (103, 104). Concurrently, depletion of extracellular ATP reduces pro-inflammatory signaling, further dampening immune responses (105, 106). In addition, Tregs influence local amino acid metabolism, for example by promoting indoleamine 2,3-dioxygenase (IDO) activity in dendritic cells, resulting in tryptophan depletion and accumulation of immunosuppressive metabolites such as kynurenine, which inhibit effector T-cell proliferation and promote immune tolerance (107–109). Cytokine-mediated inhibition further strengthens Treg-driven immunosuppression. Tregs secrete several inhibitory cytokines, including IL-10, TGF-β, and IL-35. IL-10 suppresses dendritic-cell maturation, decreases antigen presentation, and reduces the production of pro-inflammatory cytokines. TGF-β inhibits cytotoxic T lymphocyte and NK-cell activity, promotes Treg differentiation, and may directly enhance tumor invasion by supporting epithelial–mesenchymal transition and stromal remodeling (110). IL-35 can directly inhibit effector T-cell proliferation and contribute to the expansion of additional regulatory immune populations (111). Together, cytokines create a self-reinforcing suppressive network that limits antitumor immunity while supporting tumor progression. Tregs modulate immune responses through indirect regulation of other immune cell populations. They influence macrophage polarization toward an M2-like phenotype, suppress dendritic cell maturation, and inhibit NK cell cytotoxicity, thereby amplifying immunosuppressive networks within the tumor microenvironment (112, 113). Furthermore, Tregs interact with stromal and tumor cells to promote angiogenesis, extracellular matrix remodeling, and metastatic dissemination, highlighting their multifaceted role in tumor progression (114, 115). In sum, Tregs employ a multilayered and highly coordinated network of suppressive mechanisms that target immune activation at the levels of antigen presentation, signal transduction, metabolic regulation, and cytokine signaling.

## 4 Tregs in immunotherapy for breast cancer

Immunomolecular targeted therapies have emerged as a pivotal modality in breast cancer treatment, complementing conventional surgery, endocrine therapy, radiotherapy, and chemotherapy (116, 117). These strategies aim to dismantle Treg-mediated immunosuppression by attenuating Treg function, disrupting their differentiation and trafficking, and interfering with checkpoint signaling, thereby restoring effector T-cell and NK-cell activity within the tumor microenvironment (118–120). Current approaches encompass immune checkpoint blockade (anti-CTLA-4, anti-PD-1/PD-L1), depletion antibodies (anti-CD25, anti-CCR4), FoxP3-based vaccines, Toll-like receptor agonists, and metabolic modulators such as fludarabine (81, 121–123). Notably, combinatorial regimens—such as anti-CTLA-4 with low-dose IL-2 or daclizumab with tumor vaccines—synergistically suppress Tregs while amplifying CD8^+^ T-cell responses (124, 125).

**Figure 2:**
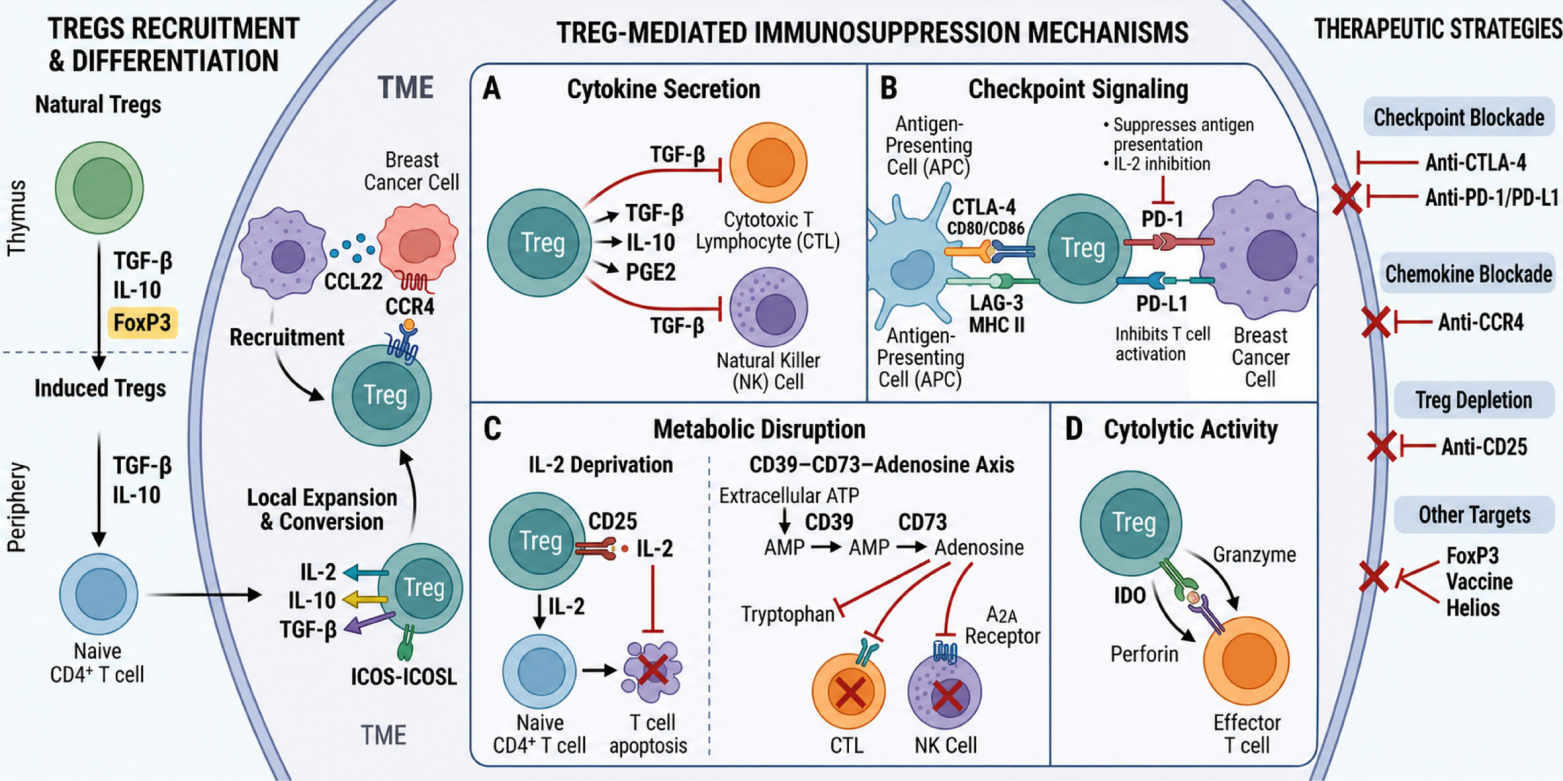
Regulatory T cells drive immune suppression in breast cancer. **(A)** Cytokine secretion. **(B)** Checkpoint signaling. **(C)** Metabolic disruption. **(D)** Cytolytic activity.

Preclinical and translational studies provide mechanistic validation for these strategies. In murine breast cancer models, miR-126-mediated inhibition of the PI3K/Akt pathway reduces Treg suppressive capacity and downregulates CTLA-4 and GITR expression (126), while anti-CCR4 antibodies selectively deplete tumor-infiltrating CCR4^+^ Tregs to potentiate antitumor immunity (123, 124). Clinically, elevated FoxP3^+^ Treg infiltration correlates with adverse prognosis yet paradoxically associates with increased cytotoxic T lymphocyte abundance, reflecting a dynamic immune equilibrium (127). Peptide vaccines targeting PD-L1 and mam-a gene therapy further demonstrate that modulating the PD-1/PD-L1 axis or reshaping CD4^+^ T-cell subsets can concurrently diminish Treg activity and enhance effector responses (128), supporting the integration of Treg-focused strategies into personalized immunotherapy frameworks (**Figure 2**).

Nevertheless, therapeutic targeting of Tregs requires precise contextual consideration due to their dual roles in tumor promotion and inflammation control. For instance, induced Tregs may attenuate tumor-associated inflammation via prostaglandin E2 secretion, potentially limiting disease progression in highly inflammatory microenvironments (129, 130). The transcription factor Helios, enriched in thymus-derived or stable suppressive Treg subsets, exemplifies this complexity: while Helios^high^ Tregs exhibit stable suppressive function and correlate with poor prognosis, Helios deficiency can destabilize Treg identity and promote conversion toward effector-like phenotypes (131). Consequently, future strategies need to balance selective depletion of tumor-promoting Treg subsets with preservation of systemic immune homeostasis, leveraging biomarkers such as Helios, CCR8, or epigenetic signatures to guide patient stratification and combination therapy design.

## 5 Conclusion

Tregs represent pivotal mediators of immune evasion in breast cancer, orchestrating a multifaceted immunosuppressive network through cytokine secretion, checkpoint engagement, metabolic reprogramming, and direct cytotoxicity. Their preferential accumulation within tumor tissues and peripheral circulation—driven by chemokine signaling, local expansion, and phenotypic conversion—consistently correlates with advanced disease stage, metastatic propensity, and unfavorable clinical outcomes. Importantly, the functional heterogeneity of tumor-infiltrating Tregs, encompassing thymus-derived, peripherally induced, and tumor-adapted subsets, underscores their context-dependent roles in shaping antitumor immunity, therapeutic resistance, and disease progression across breast cancer molecular subtypes, particularly in aggressive triple-negative disease.

Therapeutic targeting of Tregs holds considerable promise for restoring antitumor immunity, yet necessitates precision to avoid disrupting systemic immune homeostasis or provoking autoimmune toxicities. Emerging strategies—including immune checkpoint blockade, CCR4-directed depletion, FoxP3-based vaccines, and epigenetic or metabolic modulators—demonstrate potential to selectively attenuate tumor-promoting Treg subsets while preserving protective regulatory functions. Future advances will depend on integrating single-cell profiling, spatial transcriptomics, and functional biomarkers to guide patient stratification and rational combination regimens with chemotherapy, endocrine therapy, or radiotherapy. Ultimately, deciphering the plasticity, niche-specific adaptations, and lineage stability of Tregs will be essential for developing safe, effective immunotherapies that convert the immunosuppressive breast cancer microenvironment into a terrain permissive for durable, immune-mediated tumor control and improved patient survival.

## References

[1] Force LM Kocarnik JM May ML Bhangdia K Crist Penberthy L . The global, regional, and national burden of cancer, 1990-2023, with forecasts to 2050: a systematic analysis for the Global Burden of Disease Study 2023. Lancet. (2025) 406:1565–86. doi: 10.1016/s0140-6736(25)01635-6. PMID: 41015051 PMC12687902

[2] Sun J Ren S Zhao Q He J Wang Y Ren M . Endostatin-based anti-angiogenic therapy and immune modulation: mechanisms and synergistic potential in cancer treatment. Front Immunol. (2025) 16:1623859. doi: 10.3389/fimmu.2025.1623859. PMID: 40574866 PMC12198163

[3] Shichkin VP Morva A Ulaganathan VK Erin N Nativi C Brezar SK . Breast cancer immunotherapy: mechanisms of immune evasion, biomarkers, and emerging therapeutic strategies. Mol Cancer. (2026). doi: 10.1186/s12943-026-02655-0. PMID: 42035063 PMC13251094

[4] Shao Z Wang X Zhu H Yao L Zhang Q Zhai Z . Stress-programmed immune niches fuel TNFR2+ Treg activation and drive neoadjuvant chemotherapy resistance in breast cancer. Adv Sci (Weinh). (2026) 13:e12952. doi: 10.1002/advs.202512952. PMID: 41486103 PMC12970206

[5] Li N Tian D . Targeting tumor-infiltrating regulatory T cells based on immunometabolism. Cancer Biol Med. (2026) 23:186–200. doi: 10.20892/j.issn.2095-3941.2025.0645. PMID: 41814664 PMC12980038

[6] Kim N Na S Lee HJ Yi W Son GW Park J . Integrative multi-omics stratification and translational evaluation of Treg-targeted combination immunotherapy in breast cancer. Front Oncol. (2025) 15:1731411. doi: 10.3389/fonc.2025.1731411. PMID: 41568381 PMC12815797

[7] Zhang H Felthaus O Eigenberger A Klein S Prantl L . Treg cell therapeutic strategies for breast cancer: holistic to local aspects. Cells. (2024) 13:1526. doi: 10.3390/cells13181526. PMID: 39329710 PMC11429654

[8] Xu A Ayoub S Zhang H Wu Y Rau M Ma X . Regulatory T cells in invasive breast cancer: prognosis, mechanisms and therapy. Cancers (Basel). (2025) 17:3172. doi: 10.3390/cancers17193172. PMID: 41097701 PMC12524184

[9] Huang P Zhou X Zheng M Yu Y Jin G Zhang S . Regulatory T cells are associated with the tumor immune microenvironment and immunotherapy response in triple-negative breast cancer. Front Immunol. (2023) 14:1263537. doi: 10.3389/fimmu.2023.1263537. PMID: 37767092 PMC10521732

[10] Fusco C Di Rella F Liotti A Colamatteo A Ferrara AL Gigantino V . CD4(+)FOXP3Exon2(+) regulatory T cell frequency predicts breast cancer prognosis and survival. Sci Adv. (2025) 11:eadr7934. doi: 10.1126/sciadv.adr7934 39813341 PMC11734725

[11] Sarkar T Dhar S Chakraborty D Pati S Bose S Panda AK . FOXP3/HAT1 axis controls Treg infiltration in the tumor microenvironment by inducing CCR4 expression in breast cancer. Front Immunol. (2022) 13:740588. doi: 10.3389/fimmu.2022.740588. PMID: 35222362 PMC8863663

[12] Fattori S Roux H Connen E Robert L Gorvel L Le Roy A . Therapeutic targeting of tumor-infiltrating regulatory T cells in breast cancer. Cancer Res. (2022) 82:3868–79. doi: 10.1158/0008-5472.can-22-0654. PMID: 36040356

[13] Shevach EM Thornton AM . tTregs, pTregs, and iTregs: similarities and differences. Immunol Rev. (2014) 259:88–102. doi: 10.1111/imr.12160. PMID: 24712461 PMC3982187

[14] Zhang A Fan T Liu Y Yu G Li C Jiang Z . Regulatory T cells in immune checkpoint blockade antitumor therapy. Mol Cancer. (2024) 23:251. doi: 10.1186/s12943-024-02156-y. PMID: 39516941 PMC11545879

[15] Qin D Zhang Y Shu P Lei Y Li X Wang Y . Targeting tumor-infiltrating tregs for improved antitumor responses. Front Immunol. (2024) 15:1325946. doi: 10.3389/fimmu.2024.1325946. PMID: 38500876 PMC10944859

[16] Attridge K Walker LS . Homeostasis and function of regulatory T cells (Tregs) *in vivo*: lessons from TCR-transgenic Tregs. Immunol Rev. (2014) 259:23–39. doi: 10.1111/imr.12165. PMID: 24712457 PMC4237543

[17] Dikiy S Rudensky AY . Principles of regulatory T cell function. Immunity. (2023) 56:240–55. doi: 10.1016/j.immuni.2023.01.004. PMID: 36792571

[18] Sakaguchi S Kawakami R Mikami N . Treg-based immunotherapy for antigen-specific immune suppression and stable tolerance induction: a perspective. Immunother Adv. (2023) 3:ltad007. doi: 10.1093/immadv/ltad007. PMID: 37397971 PMC10309084

[19] Blinova VG Zhdanov DD . Many faces of regulatory T cells: heterogeneity or plasticity? Cells. (2024) 13:959. doi: 10.3390/cells13110959. PMID: 38891091 PMC11171907

[20] Zou Y Ye F Kong Y Hu X Deng X Xie J . The single-cell landscape of intratumoral heterogeneity and the immunosuppressive microenvironment in liver and brain metastases of breast cancer. Adv Sci (Weinh). (2023) 10:e2203699. doi: 10.1002/advs.202203699. PMID: 36529697 PMC9929130

[21] Xu L Saunders K Huang SP Knutsdottir H Martinez-Algarin K Terrazas I . A comprehensive single-cell breast tumor atlas defines epithelial and immune heterogeneity and interactions predicting anti-PD-1 therapy response. Cell Rep Med. (2024) 5:101511. doi: 10.1016/j.xcrm.2024.101511. PMID: 38614094 PMC11148512

[22] Wang Y Huang T Gu J Lu L . Targeting the metabolism of tumor-infiltrating regulatory T cells. Trends Immunol. (2023) 44:598–612. doi: 10.1016/j.it.2023.06.001. PMID: 37442660

[23] Obradovic A Ager C Turunen M Nirschl T Khosravi-Maharlooei M Iuga A . Systematic elucidation and pharmacological targeting of tumor-infiltrating regulatory T cell master regulators. Cancer Cell. (2023) 41:933–949.e11. doi: 10.1101/2022.02.22.481404. PMID: 37116491 PMC10193511

[24] Arnouk SM Kancheva D Van Damme H Courtoy GE Mora Barthelmess R Van Craenenbroeck J . Depleting IL1R2+ tumor-infiltrating regulatory T cells with an ADCC-prone nanobody construct boosts the efficacy of anti-PD-1 immunotherapy. Cancer Res. (2025) 85:4681–700. doi: 10.1158/0008-5472.can-24-3095. PMID: 40960523 PMC12666320

[25] Kidani Y Nogami W Yasumizu Y Kawashima A Tanaka A Sonoda Y . CCR8-targeted specific depletion of clonally expanded Treg cells in tumor tissues evokes potent tumor immunity with long-lasting memory. Proc Natl Acad Sci USA. (2022) 119:e2114282119. doi: 10.1073/pnas.2114282119. PMID: 35140181 PMC8851483

[26] Goral A Firczuk M Fidyt K Sledz M Simoncello F Siudakowska K . A specific CD44lo CD25lo subpopulation of regulatory T cells inhibits anti-leukemic immune response and promotes the progression in a mouse model of chronic lymphocytic leukemia. Front Immunol. (2022) 13:781364. doi: 10.3389/fimmu.2022.781364. PMID: 35296093 PMC8918500

[27] Serrano García L Jávega B Llombart Cussac A Gión M Pérez-García JM Cortés J . Patterns of immune evasion in triple-negative breast cancer and new potential therapeutic targets: a review. Front Immunol. (2024) 15:1513421. doi: 10.3389/fimmu.2024.1513421. PMID: 39735530 PMC11671371

[28] Trujillo-Ochoa JL Kazemian M Afzali B . The role of transcription factors in shaping regulatory T cell identity. Nat Rev Immunol. (2023) 23:842–56. doi: 10.1038/s41577-023-00893-7. PMID: 37336954 PMC10893967

[29] Golzari-Sorkheh M Zúñiga-Pflücker JC . Development and function of FOXP3+ regulators of immune responses. Clin Exp Immunol. (2023) 213:13–22. doi: 10.1093/cei/uxad048. PMID: 37085947 PMC10324550

[30] Yue Y Ren Y Lu C Li P Zhang G . Epigenetic regulation of human FOXP3+ Tregs: from homeostasis maintenance to pathogen defense. Front Immunol. (2024) 15:1444533. doi: 10.3389/fimmu.2024.1444533. PMID: 39144146 PMC11323565

[31] Zong Y Deng K Chong WP . Regulation of Treg cells by cytokine signaling and co-stimulatory molecules. Front Immunol. (2024) 15:1387975. doi: 10.3389/fimmu.2024.1387975. PMID: 38807592 PMC11131382

[32] Wang J Zhao X Wan YY . Intricacies of TGF-β signaling in Treg and Th17 cell biology. Cell Mol Immunol. (2023) 20:1002–22. doi: 10.1038/s41423-023-01036-7. PMID: 37217798 PMC10468540

[33] Liu J Zhang B Zhang G Shang D . Reprogramming of regulatory T cells in inflammatory tumor microenvironment: can it become immunotherapy turning point? Front Immunol. (2024) 15:1345838. doi: 10.3389/fimmu.2024.1345838. PMID: 38449875 PMC10915070

[34] Sun Q Barrett AK Ogishi M Lyu H Jiang H Liu H . Facile induction of immune tolerance by an interleukin-2-TGFβ surrogate agonist. Nature. (2026). doi: 10.1038/s41586-026-10208-0. PMID: 41813890 PMC13190267

[35] Guo H Lu L Wang R Perez-Gutierrez A Abdulkerim HS Zahorchak AF . Impact of human mutant TGFβ1/Fc protein on memory and regulatory T cell homeostasis following lymphodepletion in nonhuman primates. Am J Transplant. (2016) 16:2994–3006. doi: 10.1111/ajt.13883. PMID: 27217298 PMC5121100

[36] Sauer S Bruno L Hertweck A Finlay D Leleu M Spivakov M . T cell receptor signaling controls Foxp3 expression via PI3K, Akt, and mTOR. Proc Natl Acad Sci USA. (2008) 105:7797–802. doi: 10.1073/pnas.0800928105. PMID: 18509048 PMC2409380

[37] Kurebayashi Y Baba Y Minowa A Nadya NA Azuma M Yoshimura A . TGF-β-induced phosphorylation of Akt and Foxo transcription factors negatively regulates induced regulatory T cell differentiation. Biochem Biophys Res Commun. (2016) 480:114–9. doi: 10.1016/j.bbrc.2016.09.153. PMID: 27697523

[38] Lee J Kim D Min B . Tissue resident Foxp3(+) regulatory T cells: sentinels and saboteurs in health and disease. Front Immunol. (2022) 13:865593. doi: 10.3389/fimmu.2022.865593. PMID: 35359918 PMC8963273

[39] Guan R Pan M Xu X Du L Rao X Fu G . Interleukin-33 potentiates TGF-β signaling to regulate intestinal stem cell regeneration after radiation injury. Cell Transplant. (2023) 32:9636897231177377. doi: 10.1177/09636897231177377. PMID: 37291802 PMC10272662

[40] Fan G Zuo S Wang Z Zhang S Liu L Luo H . Targeting of the IL-33/Wnt axis restricts breast cancer stemness and metastasis. Sci Rep. (2025) 15:18172. doi: 10.1038/s41598-025-03260-9. PMID: 40414980 PMC12104483

[41] Lei S Jin J Zhao X Zhou L Qi G Yang J . The role of IL-33/ST2 signaling in the tumor microenvironment and Treg immunotherapy. Exp Biol Med (Maywood). (2022) 247:1810–8. doi: 10.1177/15353702221102094. PMID: 35733343 PMC9679353

[42] Grover P Goel PN Greene MI . Regulatory T cells: regulation of identity and function. Front Immunol. (2021) 12:750542. doi: 10.3389/fimmu.2021.750542. PMID: 34675933 PMC8524049

[43] Alvarez F Liu Z Bay A Piccirillo CA . Deciphering the developmental trajectory of tissue-resident Foxp3(+) regulatory T cells. Front Immunol. (2024) 15:1331846. doi: 10.3389/fimmu.2024.1331846. PMID: 38605970 PMC11007185

[44] Kressler C Gasparoni G Nordström K Hamo D Salhab A Dimitropoulos C . Targeted de-methylation of the FOXP3-TSDR is sufficient to induce physiological FOXP3 expression but not a functional Treg phenotype. Front Immunol. (2020) 11:609891. doi: 10.3389/fimmu.2020.609891. PMID: 33488615 PMC7817622

[45] Tian M Hao F Wang X Zheng X Wang H Li J . OGG1 augments the transcriptional activation of Foxp3 to promote iTreg differentiation for IBD alleviation. Proc Natl Acad Sci USA. (2025) 122:e2424733122. doi: 10.1073/pnas.2424733122. PMID: 40694333 PMC12318175

[46] Mikami N Kawakami R Chen KY Sugimoto A Ohkura N Sakaguchi S . Epigenetic conversion of conventional T cells into regulatory T cells by CD28 signal deprivation. Proc Natl Acad Sci USA. (2020) 117:12258–68. doi: 10.1073/pnas.1922600117. PMID: 32414925 PMC7275710

[47] Sasidharan Nair V Song MH Oh KI . Vitamin C facilitates demethylation of the Foxp3 enhancer in a Tet-dependent manner. J Immunol. (2016) 196:2119–31. doi: 10.4049/jimmunol.1502352. PMID: 26826239

[48] He M Zong X Xu B Qi W Huang W Djekidel MN . Dynamic Foxp3-chromatin interaction controls tunable Treg cell function. J Exp Med. (2024) 221:e20232068. doi: 10.1084/jem.20232068. PMID: 38935023 PMC11211070

[49] Clay R Li K Jin L . Metabolic signaling in the tumor microenvironment. Cancers (Basel). (2025) 17:155. doi: 10.3390/cancers17010155. PMID: 39796781 PMC11719658

[50] Otterlei Fjørtoft M Huse K Rye IH . The tumor immune microenvironment in breast cancer progression. Acta Oncol. (2024) 63:359–67. doi: 10.2340/1651-226x.2024.33008. PMID: 38779867 PMC11332517

[51] Muteeb G Khafaga DSR El-Morsy MT Farhan M Aatif M Hosney M . Targeting tumor-associated macrophages with nanocarrier-based treatment for breast cancer: A step toward developing innovative anti-cancer therapeutics. Heliyon. (2024) 10:e37217. doi: 10.1016/j.heliyon.2024.e37217. PMID: 39309874 PMC11415663

[52] Retecki K Seweryn M Graczyk-Jarzynka A Bajor M . The immune landscape of breast cancer: Strategies for overcoming immunotherapy resistance. Cancers (Basel). (2021) 13:6012. doi: 10.3390/cancers13236012. PMID: 34885122 PMC8657247

[53] Li J Wang S Wang N Zheng Y Yang B Wang X . Aiduqing formula inhibits breast cancer metastasis by suppressing TAM/CXCL1-induced Treg differentiation and infiltration. Cell Commun Signal. (2021) 19:89. doi: 10.1186/s12964-021-00775-2. PMID: 34461944 PMC8404313

[54] Gobert M Treilleux I Bendriss-Vermare N Bachelot T Goddard-Leon S Arfi V . Regulatory T cells recruited through CCL22/CCR4 are selectively activated in lymphoid infiltrates surrounding primary breast tumors and lead to an adverse clinical outcome. Cancer Res. (2009) 69:2000–9. doi: 10.1158/0008-5472.can-08-2360. PMID: 19244125

[55] Kuehnemuth B Piseddu I Wiedemann GM Lauseker M Kuhn C Hofmann S . CCL1 is a major regulatory T cell attracting factor in human breast cancer. BMC Cancer. (2018) 18:1278. doi: 10.1186/s12885-018-5117-8. PMID: 30572845 PMC6302432

[56] Facciabene A Peng X Hagemann IS Balint K Barchetti A Wang LP . Tumor hypoxia promotes tolerance and angiogenesis via CCL28 and T(reg) cells. Nature. (2011) 475:226–30. doi: 10.1038/nature10169. PMID: 21753853

[57] Ren L Yu Y Wang L Zhu Z Lu R Yao Z . Hypoxia-induced CCL28 promotes recruitment of regulatory T cells and tumor growth in liver cancer. Oncotarget. (2016) 7:75763–73. doi: 10.18632/oncotarget.12409. PMID: 27716621 PMC5342776

[58] Mergia Terefe E Catalan Opulencia MJ Rakhshani A Ansari MJ Sergeevna SE Awadh SA . Roles of CCR10/CCL27-CCL28 axis in tumor development: mechanisms, diagnostic and therapeutic approaches, and perspectives. Expert Rev Mol Med. (2022) 24:e37. doi: 10.1017/erm.2022.28. PMID: 36155126

[59] Boeker V Kalluri R . Insights into the relevance of targeting fibroblasts to control cancer. Cell Rep Med. (2025) 6:102395. doi: 10.1016/j.xcrm.2025.102395. PMID: 41187743 PMC12711671

[60] Li Y Wang C Huang T Yu X Tian B . The role of cancer-associated fibroblasts in breast cancer metastasis. Front Oncol. (2023) 13:1194835. doi: 10.3389/fonc.2023.1194835. PMID: 37496657 PMC10367093

[61] Koppensteiner L Mathieson L O'Connor RA Akram AR . Cancer associated fibroblasts - An impediment to effective anti-cancer T cell immunity. Front Immunol. (2022) 13:887380. doi: 10.3389/fimmu.2022.887380. PMID: 35479076 PMC9035846

[62] Hinton CV Avraham S Avraham HK . Role of the CXCR4/CXCL12 signaling axis in breast cancer metastasis to the brain. Clin Exp Metastasis. (2010) 27:97–105. doi: 10.1007/s10585-008-9210-2. PMID: 18814042

[63] Malla RR Vasudevaraju P Vempati RK Rakshmitha M Merchant N Nagaraju GP . Regulatory T cells: Their role in triple-negative breast cancer progression and metastasis. Cancer. (2022) 128:1171–83. doi: 10.1002/cncr.34084. PMID: 34990009

[64] Pan Y Zhou H Sun Z Zhu Y Zhang Z Han J . Regulatory T cells in solid tumor immunotherapy: effect, mechanism and clinical application. Cell Death Dis. (2025) 16:277. doi: 10.1038/s41419-025-07544-w. PMID: 40216744 PMC11992189

[65] Shouse AN LaPorte KM Malek TR . Interleukin-2 signaling in the regulation of T cell biology in autoimmunity and cancer. Immunity. (2024) 57:414–28. doi: 10.1016/j.immuni.2024.02.001. PMID: 38479359 PMC11126276

[66] Schroth SL Zhang L Jones RT Glinton K Mani NL Inui H . Treg activation during allograft tolerance induction requires mitochondrion-induced TGF-β1 in type 1 conventional dendritic cells. J Clin Invest. (2025) 135:e178960. doi: 10.1172/jci178960. PMID: 40644411 PMC12435855

[67] Xu L Xu W Qiu S Xiong S . Enrichment of CCR6+Foxp3+ regulatory T cells in the tumor mass correlates with impaired CD8+ T cell function and poor prognosis of breast cancer. Clin Immunol. (2010) 135:466–75. doi: 10.1016/j.clim.2010.01.014. PMID: 20181533

[68] Deng S Chen Y Song B Wang H Huang S Wu K . Tertiary lymphoid structures in cancer: spatiotemporal heterogeneity, immune orchestration, and translational opportunities​​. J Hematol Oncol. (2025) 18:97. doi: 10.1186/s13045-025-01754-7. PMID: 41219991 PMC12606831

[69] Tiwari PK Chaudhary AA Gupta S Chouhan M Singh HN Rustagi S . Extracellular vesicles in triple-negative breast cancer: current updates, challenges and future prospects. Front Mol Biosci. (2025) 12:1561464. doi: 10.3389/fmolb.2025.1561464. PMID: 40297849 PMC12034555

[70] Ma C Teoh HK Zhao Y Wang Y Zhao J Liu Y . Reprogramming the immunosuppressive breast cancer microenvironment: integrating cellular, metabolic, and stromal targets for rational immunotherapy. Front Immunol. (2026) 17:1760782. doi: 10.3389/fimmu.2026.1760782. PMID: 41766877 PMC12946151

[71] Ma N Chen T Zhang Y Chen L Li J Peng X . ICOSL expressed in triple-negative breast cancer can induce Foxp3+ Treg cell differentiation and reverse p38 pathway activation. Am J Cancer Res. (2022) 12:4177–95. 36225638 PMC9548022

[72] Nikanjam M Kato S Nishizaki D Miyashita H Pabla S Nesline MK . Inducible T-cell co-stimulator (ICOS) and ICOS ligand: dealing with a two-faced cancer immunoregulatory system. Cancer Med. (2026) 15:e71467. doi: 10.1002/cam4.71467 41474629 PMC12755401

[73] Ma J Chen Y Li T Cao Y Hu B Liu Y . Suppression of lysosome metabolism-meditated GARP/TGF-β1 complexes specifically depletes regulatory T cells to inhibit breast cancer metastasis. Oncogene. (2024) 43:1930–40. doi: 10.1038/s41388-024-03043-y. PMID: 38698265

[74] Jiang P Li X Wang Z Li S Huang Y Li YX . COL3A1(high) cancer-associated fibroblasts orchestrate metabolic and immune microenvironments to confer chemoresistance in breast cancer. NPJ Precis Oncol. (2026) 10:139. doi: 10.1038/s41698-026-01338-9. PMID: 41731102 PMC13039519

[75] Jadidi-Niaragh F Atyabi F Rastegari A Kheshtchin N Arab S Hassannia H . CD73 specific siRNA loaded chitosan lactate nanoparticles potentiate the antitumor effect of a dendritic cell vaccine in 4T1 breast cancer bearing mice. J Control Release. (2017) 246:46–59. doi: 10.1016/j.jconrel.2016.12.012. PMID: 27993599

[76] Schreier A Zappasodi R Serganova I Brown KA Demaria S Andreopoulou E . Facts and perspectives: Implications of tumor glycolysis on immunotherapy response in triple negative breast cancer. Front Oncol. (2022) 12:1061789. doi: 10.3389/fonc.2022.1061789. PMID: 36703796 PMC9872136

[77] Peralta RM Xie B Lontos K Nieves-Rosado H Spahr K Joshi S . Dysfunction of exhausted T cells is enforced by MCT11-mediated lactate metabolism. Nat Immunol. (2024) 25:2297–307. doi: 10.4049/jimmunol.212.supp.1394.5643 PMC11588660 39516648

[78] Jeon SH Suh KJ Jung S Jeon M Jang HC Kim ES . Proliferation of tumor-related regulatory T cells in circulation dictates efficacy of chemoimmunotherapy in triple-negative breast cancer. Clin Cancer Res. (2025) 31:4586–97. doi: 10.1158/1078-0432.ccr-24-3283. PMID: 40833777

[79] Watson MJ Vignali PDA Mullett SJ Overacre-Delgoffe AE Peralta RM Grebinoski S . Metabolic support of tumor-infiltrating regulatory T cells by lactic acid. Nature. (2021) 591:645–51. doi: 10.1038/s41586-020-03045-2. PMID: 33589820 PMC7990682

[80] Bui TM Jimenez ER Li Z Foidart P Puleo J Yan P . Identification of cycling regulatory T cell precursors as conductors of immune escape during breast carcinoma progression. Cancer Cell. (2026). doi: 10.1016/j.ccell.2026.03.015. PMID: 41997138 PMC13213619

[81] Fattori S Le Roy A Houacine J Robert L Abes R Gorvel L . CD25high effector regulatory T cells hamper responses to PD-1 blockade in triple-negative breast cancer. Cancer Res. (2023) 83:3026–44. doi: 10.1158/0008-5472.can-23-0613. PMID: 37379438 PMC10502453

[82] Zitti B Duval F Wirapati P Hicham M Xie Y Oh J . Positioning and reversible suppression of CCR7(+) dendritic cells in perivascular tumor niches shape cancer immunity. Immunity. (2026) 59:161–176.e12. doi: 10.1016/j.immuni.2025.11.020. PMID: 41421339 PMC12882814

[83] Si F Liu X Tao Y Zhang Y Ma F Hsueh EC . Blocking senescence and tolerogenic function of dendritic cells induced by γδ Treg cells enhances tumor-specific immunity for cancer immunotherapy. J Immunother Cancer. (2024) 12:e008219. doi: 10.1136/jitc-2023-008219. PMID: 38580332 PMC11002396

[84] Pei X Wang X Li H . LncRNA SNHG1 regulates the differentiation of Treg cells and affects the immune escape of breast cancer via regulating miR-448/IDO. Int J Biol Macromol. (2018) 118:24–30. doi: 10.1016/j.ijbiomac.2018.06.033. PMID: 29886172

[85] Olkhanud PB Damdinsuren B Bodogai M Gress RE Sen R Wejksza K . Tumor-evoked regulatory B cells promote breast cancer metastasis by converting resting CD4^+^ T cells to T-regulatory cells. Cancer Res. (2011) 71:3505–15. doi: 10.1158/0008-5472.can-10-4316. PMID: 21444674 PMC3096701

[86] Wang R Huang K . CCL11 increases the proportion of CD4+CD25+Foxp3+ Treg cells and the production of IL-2 and TGF-β by CD4+ T cells via the STAT5 signaling pathway. Mol Med Rep. (2020) 21:2522–32. doi: 10.3892/mmr.2020.11049. PMID: 32323817 PMC7185287

[87] Cao X Cai SF Fehniger TA Song J Collins LI Piwnica-Worms DR . Granzyme B and perforin are important for regulatory T cell-mediated suppression of tumor clearance. Immunity. (2007) 27:635–46. doi: 10.1016/j.immuni.2007.08.014. PMID: 17919943

[88] Sula Karreci E Eskandari SK Dotiwala F Routray SK Kurdi AT Assaker JP . Human regulatory T cells undergo self-inflicted damage via granzyme pathways upon activation. JCI Insight. (2017) 2:e91599. doi: 10.1172/jci.insight.91599. PMID: 29093262 PMC5690280

[89] Wang A Wang Y Liang R Li B Pan F . Improving regulatory T cell-based therapy: insights into post-translational modification regulation. J Genet Genomics. (2025) 52:145–56. doi: 10.1016/j.jgg.2024.09.014. PMID: 39357622

[90] Kos K de Visser KE . The multifaceted role of regulatory T cells in breast cancer. Annu Rev Cancer Biol. (2021) 5:291–310. doi: 10.1146/annurev-cancerbio-042920-104912. PMID: 34632244 PMC7611782

[91] Yang Y Wang W . Recent progress in immune evasion mechanisms of triple-negative breast cancer. J Transl Med. (2025) 23:1314. doi: 10.1186/s12967-025-07370-w. PMID: 41254706 PMC12624998

[92] Xie J Hamdy H Elkord E . Treg-directed cancer immunotherapy beyond immune checkpoints: progress and opportunities. Trends Pharmacol Sci. (2026). doi: 10.1016/j.tips.2026.03.007. PMID: 42049529

[93] Zappasodi R Serganova I Cohen IJ Maeda M Shindo M Senbabaoglu Y . CTLA-4 blockade drives loss of T(reg) stability in glycolysis-low tumors. Nature. (2021) 591:652–8. doi: 10.1038/s41586-021-03326-4. PMID: 33588426 PMC8057670

[94] Blomberg OS Kos K Spagnuolo L Isaeva OI Garner H Wellenstein MD . Neoadjuvant immune checkpoint blockade triggers persistent and systemic T(reg) activation which blunts therapeutic efficacy against metastatic spread of breast tumors. Oncoimmunology. (2023) 12:2201147. doi: 10.1080/2162402x.2023.2201147. PMID: 37089449 PMC10114978

[95] Wing JB Ise W Kurosaki T Sakaguchi S . Regulatory T cells control antigen-specific expansion of Tfh cell number and humoral immune responses via the coreceptor CTLA-4. Immunity. (2014) 41:1013–25. doi: 10.1016/j.immuni.2014.12.006. PMID: 25526312

[96] Tekguc M Wing JB Osaki M Long J Sakaguchi S . Treg-expressed CTLA-4 depletes CD80/CD86 by trogocytosis, releasing free PD-L1 on antigen-presenting cells. Proc Natl Acad Sci USA. (2021) 118:e2023739118. doi: 10.4049/jimmunol.206.supp.96.04 34301886 PMC8325248

[97] Frijlink E Bosma DMT Busselaar J Battaglia TW Staal MD Verbrugge I . PD-1 or CTLA-4 blockade promotes CD86-driven Treg responses upon radiotherapy of lymphocyte-depleted cancer in mice. J Clin Invest. (2024) 134:e171154. doi: 10.1172/jci171154. PMID: 38349740 PMC10940086

[98] Liu R Li HF Li S . PD-1-mediated inhibition of T cell activation: Mechanisms and strategies for cancer combination immunotherapy. Cell Insight. (2024) 3:100146. doi: 10.1016/j.cellin.2024.100146. PMID: 38425643 PMC10901852

[99] Syamsu SA Faruk M Smaradania N Sampepajung E Pranoto AS Irsandy F . PD-1/PD-L1 pathway: Current research in breast cancer. Breast Dis. (2024) 43:79–92. doi: 10.3233/bd-249006. PMID: 38701137 PMC11091639

[100] Javed SA Najmi A Ahsan W Zoghebi K . Targeting PD-1/PD-L-1 immune checkpoint inhibition for cancer immunotherapy: success and challenges. Front Immunol. (2024) 15:1383456. doi: 10.3389/fimmu.2024.1383456. PMID: 38660299 PMC11039846

[101] Lei C Lu W Li Y Yang H Zhang K Wang N . Serpina1 gene regulates the tumorigenesis and progression of breast cancer through PI3K/AKT signaling pathway and tumor immune microenvironment. Sci Rep. (2025) 15:38901. doi: 10.1038/s41598-025-22658-z. PMID: 41198720 PMC12592430

[102] Li S Zhang X Pang D . Pirfenidone inhibits CCL2-mediated Treg chemotaxis induced by palbociclib and fulvestrant in HR+/HER2- breast cancer. Int Immunopharmacol. (2024) 142:113059. doi: 10.1016/j.intimp.2024.113059. PMID: 39241517

[103] Shen J Liao B Gong L Li S Zhao J Yang H . Cd39 and cd73: biological functions, diseases and therapy. Mol BioMed. (2025) 6:97. doi: 10.1186/s43556-025-00345-9. PMID: 41188606 PMC12586806

[104] Huang T Ren X Tang X Wang Y Ji R Guo Q . Current perspectives and trends of CD39-CD73-eAdo/A2aR research in tumor microenvironment: a bibliometric analysis. Front Immunol. (2024) 15:1427380. doi: 10.3389/fimmu.2024.1427380. PMID: 39188712 PMC11345151

[105] Jin JO Zhang W Wong KW Kwak M van Driel IR Yu Q . Inhibition of breast cancer resistance protein (ABCG2) in human myeloid dendritic cells induces potent tolerogenic functions during LPS stimulation. PloS One. (2014) 9:e104753. doi: 10.1371/journal.pone.0104753. PMID: 25111504 PMC4128747

[106] Jahangiri S Bourdages S Skora E Stagg J Yu F . Atp released by ultrasound targeted microbubble cavitation induces vascular inflammation and improves immune checkpoint blockade efficacy. Theranostics. (2025) 15:5220–37. doi: 10.7150/thno.105857. PMID: 40303330 PMC12036883

[107] Sarangi P . Role of indoleamine 2, 3-dioxygenase 1 in immunosuppression of breast cancer. Cancer Pathog Ther. (2024) 2:246–55. doi: 10.1016/j.cpt.2023.11.001. PMID: 39371092 PMC11447360

[108] Asghar K Loya A Rana IA Bakar MA Farooq A Tahseen M . Forkhead box P3 and indoleamine 2,3-dioxygenase co-expression in Pakistani triple negative breast cancer patients. World J Clin Oncol. (2020) 11:1018–28. doi: 10.5306/wjco.v11.i12.1018. PMID: 33437664 PMC7769718

[109] Guan J Wu Y Liu X Wang H Ye N Li Z . A novel prodrug and its nanoformulation suppress cancer stem cells by inducing immunogenic cell death and inhibiting indoleamine 2, 3-dioxygenase. Biomaterials. (2021) 279:121180. doi: 10.1016/j.biomaterials.2021.121180. PMID: 34768152

[110] Hamidinia M Ghafourian Boroujerdnia M Talaiezadeh A Solgi G Roshani R Iranprast S . Increased P-35, EBI3 transcripts and other Treg markers in peripheral blood mononuclear cells of breast cancer patients with different clinical stages. Adv Pharm Bull. (2015) 5:261–7. doi: 10.15171/apb.2015.036. PMID: 26236666 PMC4517085

[111] Khalil RG Mohammed DA Hamdalla HM Ahmed OM . The possible anti-tumor effects of regulatory T cells plasticity / IL-35 in the tumor microenvironment of the major three cancer types. Cytokine. (2025) 186:156834. doi: 10.1016/j.cyto.2024.156834. PMID: 39693872

[112] Ma Y Su H Wang X Niu X Che Y Hambly BD . The role of IL-35 and IL-37 in breast cancer - potential therapeutic targets for precision medicine. Front Oncol. (2022) 12:1051282. doi: 10.3389/fonc.2022.1051282. PMID: 36483045 PMC9723453

[113] Ge J Yin X Chen L . Regulatory T cells: masterminds of immune equilibrium and future therapeutic innovations. Front Immunol. (2024) 15:1457189. doi: 10.3389/fimmu.2024.1457189. PMID: 39290699 PMC11405253

[114] Zhou Z Zhong H Wang H Wang S Kridis WB Wang R . Microenvironmental regulation and remodeling of breast cancer angiogenesis: from basic mechanisms to clinical therapeutic implications. Discov Oncol. (2025) 16:1973. doi: 10.1007/s12672-025-03797-1. PMID: 41144119 PMC12559528

[115] Yuan Z Li Y Zhang S Wang X Dou H Yu X . Extracellular matrix remodeling in tumor progression and immune escape: from mechanisms to treatments. Mol Cancer. (2023) 22:48. doi: 10.1186/s12943-023-01744-8. PMID: 36906534 PMC10007858

[116] Harris MA Savas P Virassamy B O'Malley MMR Kay J Mueller SN . Towards targeting the breast cancer immune microenvironment. Nat Rev Cancer. (2024) 24:554–77. doi: 10.1038/s41568-024-00714-6. PMID: 38969810

[117] Rebaudi F De Franco F Goda R Obino V Vita G Baronti C . The landscape of combining immune checkpoint inhibitors with novel therapies: secret alliances against breast cancer. Cancer Treat Rev. (2024) 130:102831. doi: 10.1016/j.ctrv.2024.102831. PMID: 39342797

[118] Debesset A Pilon C Meunier S Cuelenaere-Bonizec O Richer W Thiolat A . Tnfr2 blockade promotes antitumoral immune response in PDAC by targeting activated Treg and reducing T cell exhaustion. J Immunother Cancer. (2024) 12:e008898. doi: 10.1136/jitc-2024-008898. PMID: 39562007 PMC11580249

[119] Kos K Aslam MA van de Ven R Wellenstein MD Pieters W van Weverwijk A . Tumor-educated T(regs) drive organ-specific metastasis in breast cancer by impairing NK cells in the lymph node niche. Cell Rep. (2022) 38:110447. doi: 10.1016/j.celrep.2022.110447. PMID: 35235800

[120] Hong H Gu Y Zhang H Simon AK Chen X Wu C . Depletion of CD4+CD25+ regulatory T cells enhances natural killer T cell-mediated anti-tumor immunity in a murine mammary breast cancer model. Clin Exp Immunol. (2010) 159:93–9. doi: 10.1111/j.1365-2249.2009.04018.x. PMID: 19817769 PMC2802699

[121] Wang X Tan B Liu J Wang J Chen M Yang Q . EChinacoside inhibits tumor immune evasion by downregulating inducible PD-L1 and reshaping tumor immune landscape in breast and colorectal cancer. Phytomedicine. (2024) 135:156188. doi: 10.1016/j.phymed.2024.156188. PMID: 39488102

[122] Jama M Tabana Y Barakat KH . Targeting cytotoxic lymphocyte antigen 4 (CTLA-4) in breast cancer. Eur J Med Res. (2024) 29:353. doi: 10.1186/s40001-024-01901-9. PMID: 38956700 PMC11218087

[123] Wang C Li J Tong Y Chang H Xu J Zhu H . Anti-HER2×CCR4 bispecific antibody enhances antitumor immunity in advanced HER2-positive tumors by chemotaxis blockade and depletion of tumor-associated Tregs, without inducing systemic toxicity. J Immunother Cancer. (2025) 13:e012829. doi: 10.1136/jitc-2025-012829. PMID: 41423265 PMC12719890

[124] Sugiyama D Nishikawa H Maeda Y Nishioka M Tanemura A Katayama I . Anti-CCR4 mAb selectively depletes effector-type FoxP3+CD4+ regulatory T cells, evoking antitumor immune responses in humans. Proc Natl Acad Sci USA. (2013) 110:17945–50. doi: 10.1073/pnas.1316796110. PMID: 24127572 PMC3816454

[125] Onda M Kobayashi K Pastan I . Depletion of regulatory T cells in tumors with an anti-CD25 immunotoxin induces CD8 T cell-mediated systemic antitumor immunity. Proc Natl Acad Sci USA. (2019) 116:4575–82. doi: 10.1073/pnas.1820388116. PMID: 30760587 PMC6410866

[126] Qin A Wen Z Zhou Y Li Y Li Y Luo J . Microrna-126 regulates the induction and function of CD4(+) Foxp3(+) regulatory T cells through PI3K/AKT pathway. J Cell Mol Med. (2013) 17:252–64. doi: 10.1111/jcmm.12003 PMC3822588 23301798

[127] Liu F Lang R Zhao J Zhang X Pringle GA Fan Y . CD8^+^ cytotoxic T cell and FOXP3^+^ regulatory T cell infiltration in relation to breast cancer survival and molecular subtypes. Breast Cancer Res Treat. (2011) 130:645–55. doi: 10.1007/s10549-011-1647-3. PMID: 21717105

[128] Tiriveedhi V Fleming TP Goedegebuure PS Naughton M Ma C Lockhart C . Mammaglobin-A cDNA vaccination of breast cancer patients induces antigen-specific cytotoxic CD4+ICOShi T cells. Breast Cancer Res Treat. (2013) 138:109–18. doi: 10.1007/s10549-012-2110-9. PMID: 22678162 PMC3656506

[129] Whiteside TL . Regulatory T cell subsets in human cancer: are they regulating for or against tumor progression? Cancer Immunol Immunother. (2014) 63:67–72. doi: 10.1007/s00262-013-1490-y. PMID: 24213679 PMC3888225

[130] Karavitis J Zhang M . Cox2 regulation of breast cancer bone metastasis. Oncoimmunology. (2013) 2:e23129. doi: 10.4161/onci.23129. PMID: 23802065 PMC3661150

[131] Santagata S Trotta AM D'Alterio C Napolitano M Rea G Di Napoli M . KIR2DL2/DL3+NKs and Helios+Tregs in peripheral blood predict nivolumab response in patients with metastatic renal cell cancer. Clin Cancer Res. (2024) 30:4755–67. doi: 10.1158/1078-0432.ccr-24-0729. PMID: 39167621 PMC11474171

